# Supplementary material for: Quantifying vector diversion effects in zoonotic systems: A modelling framework for arbovirus transmission between reservoir and dead-end hosts
Source: PLoS Comput Biol. 2025 Dec 18;21(12):e1013359. doi: 10.1371/journal.pcbi.1013359 (PMC12795452; doi:10.1371/journal.pcbi.1013359)
Supplement: S1 Text — (PDF) [file pcbi.1013359.s001.pdf]

Quantifying vector diversion effects in zoonotic systems: A modelling framework for arbovirus transmission between reservoir and dead-end hosts

Emma L Fairbanks[1,2,*], Matthew Baylis[3], Janet M Daly[4], and Michael J Tildesley[1]

[1]The Zeeman Institute for Systems Biology & Infectious Disease Epidemiology Research, Mathematics Institute and School of Life Sciences, University of Warwick, Coventry, UK
[2]Department of Mathematics, University of Manchester, Manchester, UK
[3]Institute of Infection, Veterinary and Ecological Sciences, Faculty of Health and Life Sciences, University of Liverpool, Liverpool, UK
[4]One Virology - Wolfson Centre for Global Virus Research, School of Veterinary Medicine and Science, University of Nottingham, Loughborough, UK
[*]Corresponding author: emma.fairbanks@manchester.ac.uk

1 Model development

1.1 The baseline model

The Fairbanks et al. [1] model categorises host encountering events as successful feeding, preprandial mortality or disarming. The daily probability of an host encountering event on day s follows Markovian principles, calculated as

$$P_\alpha(s) = 1 - \exp(-\epsilon\hat{\alpha}[s]\beta), \tag{1}$$

where ϵ the number of times the vector feeds per gonotrophic cycle, $\hat{\alpha}[s]$ is the rate of gonotrophic cycle completion on day s and

$$\beta = \chi\big(\psi(1 - \phi(\pi - \kappa_M - \kappa_D)) + (1 - \psi)\big) + (1 - \chi). \tag{2}$$

The composite parameter β represents the adjusted rate of host-encountering events, accounting for baseline host selection and the effects of vector control tools across all host types. Here, the parameters π, κ_M and κ_D describe the effects of the tool on feeding, preprandial mortality and disarming, respectively, as described in Table A. The impact of vector-control tools on these modes of action is scaled by their usage (ϕ), coverage (ψ) and the baseline host selection of vectors without interventions present (χ).

Given a pathogen is transmitted to a vector on day t, the probability that the vector survives until day τ is

$$P_\mu(t, \tau) = \exp\left(-\sum_{s=t}^{\tau} \hat{\mu}[s]\right), \tag{3}$$

where $\hat{\mu}[s]$ is defined as the vector mortality rate on day s.

1.2 Reformating parameters

To extend the model to consider how pathogens interact with multiple host types many of the scalar parameters in Fairbanks et al. [1] are considered to be vectors (Table A).

In the modelling framework climate-dependent variables are represented as mathematical vectors. The model can either be simulated with each vector entry representing a predicted daily value calculated from climatic data or, alternatively, treated as constants where every entry has the same value, calculated for a constant temperature.

Fairbanks et al. [1] considered the climatic dependence of the rates of gonotrophic cycle completion, vector mortality and pathogen extrinsic incubation period (EIP) completion.

1.3 Model extension

The model is updated to account for the interactions between vectors and different host types. The probability of a host encountering event is considered to follow the Markovian model described in Equation 1. However, β is modified to

$$\beta = \sum_{h=1}^{N_h} \chi[h]\Big(\psi[h]\big(1 - \phi[h](\pi[h] - \kappa_M[h] - \kappa_D[h])\big) + (1 - \psi_h)\Big) + 1 - \sum_{h=1}^{N_h} \chi_h, \tag{4}$$

where N_h is the number of host types considered.

The rates of the preprandial and postprandial killing effects of tools on any given day s due to interactions vector with host type $h \in \{1, \ldots, N_h\}$ are calculated as

$$\hat{\gamma}_{pre}[h, s] = P_\alpha(s)\frac{\chi[h]\psi[h]\phi[h]\kappa_M[h]}{\beta} \text{ and} \tag{5}$$

$$\hat{\gamma}_{post}[h, s] = P_\alpha(s)\frac{\chi[h]\psi[h](1 - \phi[h]\pi[h])\xi[h]}{\beta}, \tag{6}$$

respectively.

Now, we will consider the realtive vectorial capacity (rVC) from a reservoir host, denoted host h_R, to all host types considered in the model. As an example we will consider a host transmitted to denoted as h_E. It is important to note that h_R may be the same as h_E, that is we consider the transmission between hosts of the same type.

For simplicity, the values for parameters which describe each host type, for example π, the values corresponding to h_R and h_E are denoted $\pi[h_R]$ and $\pi[h_E]$, respectively.

The probability a bite with transmission of a pathogen from h_R to a vector and vector to h_E on days t and τ are

$$P_{h\to v}(t) = P_\alpha(t)\rho_{h\to v}[h_R]\frac{\chi[h_R]\big(\psi[h_R](1 - \phi[h_R]\pi[h_R]) + (1 - \psi[h_R])\big)}{\beta} \tag{7}$$

Table A: Fundamental parameter definitions. Parameters denoted with a ˆ are dependent on climatic conditions.

Parameter	Dimension	Definition
T	scalar	Number of days (time steps) simulated.
N_h	scalar	Number of host-types.
$\hat{\alpha}$	T	Rate of gonotrophic cycle completion $\forall s \in \{1, \ldots, T\}$.
$\hat{\mu}$	T	Vector mortality rate $\forall s \in \{1, \ldots, T\}$.
$\hat{\sigma}$	T	Rate of pathogen EIP completion $\forall s \in \{1, \ldots, T\}$.
χ	N_h	Proportion of blood meals from host type $h \in \{1, \ldots, N_h\}$, referred to as the blood index.
ϵ	scalar	Average number of bites per vector per gonotrophic cycle.
π	N_h	Reduction in the rate of vector biting on host type $h \in \{1, \ldots, N_h\}$ due to the presence of a vector- control tool.
κ_M	N_h	Increase in the rate of vector mortality before biting host type $h \in \{1, \ldots, N_h\}$ due to the presence of a vector-control tool, relative to the rate of biting host type h without the vector-control tool.
κ_D	N_h	Rate of vector disarming due to the presence of a vector-control tool when seeking to feed on host type $h \in \{1, \ldots, N_h\}$, relative to the rate of biting host type h without the vector-control tool.
ξ	N_h	Increased probability of vector mortality after biting host type $h \in \{1, \ldots, N_h\}$ due to the presence of a vector control tool.
ψ	N_h	Proportion of host type $h \in \{1, \ldots, N_h\}$ with access to the vector-control tool, referred to as coverage.
ϕ	N_h	Adherence of host type $h \in \{1, \ldots, N_h\}$ to using the vector-control tool, referred to as usage.
$\rho_{h \to v}$	N_h	Probability of transmission from host type $h \in \{1, \ldots, N_h\}$ to vector, given the host is infectious.
$\rho_{v \to h}$	N_h	Probability of transmission from vector to host type $h \in \{1, \ldots, N_h\}$, given the vector is infectious.

Table B: Composite parameter definitions. All composite parameters are scalar.

Parameter	Definition
$P_\alpha(s)$	Probability of a host-encountering event on day s (defined as feeding or preprandial mortality or disarming due to the presence of a tool).
$P_\mu(t,\tau)$	Probability a vector which fed on day t survives until day τ.
$P_\sigma(t,\tau)$	Probability that a vector which fed on day t is infectious day τ, given transmission occurred during the bite on day t.
$\hat{\gamma}_{pre}(s)$	Rate of preprandial killing on day s.
$\hat{\gamma}_{post}(s)$	Rate of postprandial killing on day s.
$P_M(t,\tau)$	Probability a vector is killed by tool by day τ given it fed on day t.

and

$$P_{v\to h}(t,\tau) = P_\alpha(\tau)P_\sigma(t,\tau)\rho_{v\to h}[h_E]\frac{\chi[h_E]\big(\psi[h_E](1-\phi[h_E]\pi[h_E]) + (1-\psi[h_E])\big)}{\beta}, \quad (8)$$

respectively.

Assuming vector mortality events due to the presence of a tool are Markovian, the probability mortality caused by a tool before a host-encountering event on day τ given it feeds on day t is

$$P_M(t,\tau) = 1 - \exp\left(\left(\sum_{\forall h \in 1,\dots,N_h}\sum_{m=t+1}^{\tau-1}\hat{\gamma}_{pre}[h,m]\right) + \left(\sum_{m=t}^{\tau-1}\hat{\gamma}_{post}[h_R,m] + \sum_{\forall h\neq h_R}\sum_{m=t+1}^{\tau-1}\hat{\gamma}_{post}[h,m]\right)\right). \quad (9)$$

Here, preprandial mortality is not considered on day τ because it is considered in the probability of encountering a host, and therefore $P_{v\to h}$. On day t, postprandial mortality is only considered for host type h_R, since this is the host type bitten on this day.

We therefore have that the rVC from host type h_C to h_E is

$$VC(t) = P_{h\to v}(t)P_\mu(t,t) \times \sum_{x=t+1}^{\infty}\Big(P_{v\to h}(t,x)P_\mu(t,x) \times \big(1 - P_M(t,x)\big)\Big). \quad (10)$$

We consider the basic reproduction number (R_0) as the product of rVC, the infectious period and the expected number of vectors per host [1].

2 Model application: West Nile virus transmission by *Culex pipiens*

2.1 Model parameterisation

The rate of gonotrophic cycle completion is calculated as

$$\hat{\alpha} = \begin{cases} 1.70 \times 10^{-4} temp(temp - 9.4)(39.6 - temp)^{1/2}, & \text{if } 9.4 < temp < 39.6, \\ 0, & \text{otherwise.} \end{cases} \tag{11}$$

where *temp* is the temperature. For the rate of mortality, we use the reciprocal of the vector lifespan parameterised by

$$\hat{\mu} = \begin{cases} 1/(124.91681 - 3.8549414 temp), & \text{if } temp < 32. \\ 0, & \text{otherwise.} \end{cases} \tag{12}$$

These were parameterised in Shocket et al. [2].

The EIR is assumed to follow a gamma distribution with a fixed shape parameter $\alpha = 1.81$ and rate $\hat{\sigma} = \alpha / f(temp)$, where

$$f(temp) = \exp(8.4312 - 0.1267285 \times temp). \tag{13}$$

This was parameterised in Vollans et al. [3]. This parameterisation includes the probability of transmission from host to vector and therefore we set $\rho_{h \to v} = 1$ for reservoir hosts. The probability of transmission from vector to host is assumed to be the same for all hosts, giving $\rho_{v \to h} = 0.74$ for all three hosts [4].

References

[1] EL Fairbanks, JM Daly, and MJ Tildesley. Modelling the influence of climate and vector control interventions on arbovirus transmission. *Viruses*, 16(8):1221, 2024. doi: 10.3390/v16081221.

[2] Marta S Shocket, Anna B Verwillow, Mailo G Numazu, Hani Slamani, Jeremy M Cohen, Fadoua El Moustaid, Jason Rohr, Leah R Johnson, and Erin A Mordecai. Transmission of West Nile and five other temperate mosquito-borne viruses peaks at temperatures between 23 c and 26 c. *Elife*, 9:e58511, 2020. doi: 10.7554/eLife.58511.

[3] M Vollans, J Day, S Cant, J Hood, AM Kilpatrick, LD Kramer, A Vaux, J Medlock, T Ward, and RS Paton. Modelling the temperature dependent extrinsic incubation period of West Nile virus using Bayesian time delay models. *J Infect*, 89(6):106296, 2024. doi: 10.1016/j.jinf.2024.106296.

[4] Marjorie J Wonham, Mark A Lewis, Joanna Rencławowicz, and P Van den Driessche. Transmission assumptions generate conflicting predictions in host–vector disease models: a case study in West Nile virus. *Ecol Lett*, 9(6):706–725, 2006. doi: 10.1111/j.1461-0248.2006.00912.x.
