# Supplementary figures and images for: Quantifying vector diversion effects in zoonotic systems: A modelling framework for arbovirus transmission between reservoir and dead-end hosts
Source: PLoS Comput Biol. 2025 Dec 18;21(12):e1013359. doi: 10.1371/journal.pcbi.1013359 (PMC12795452; doi:10.1371/journal.pcbi.1013359)

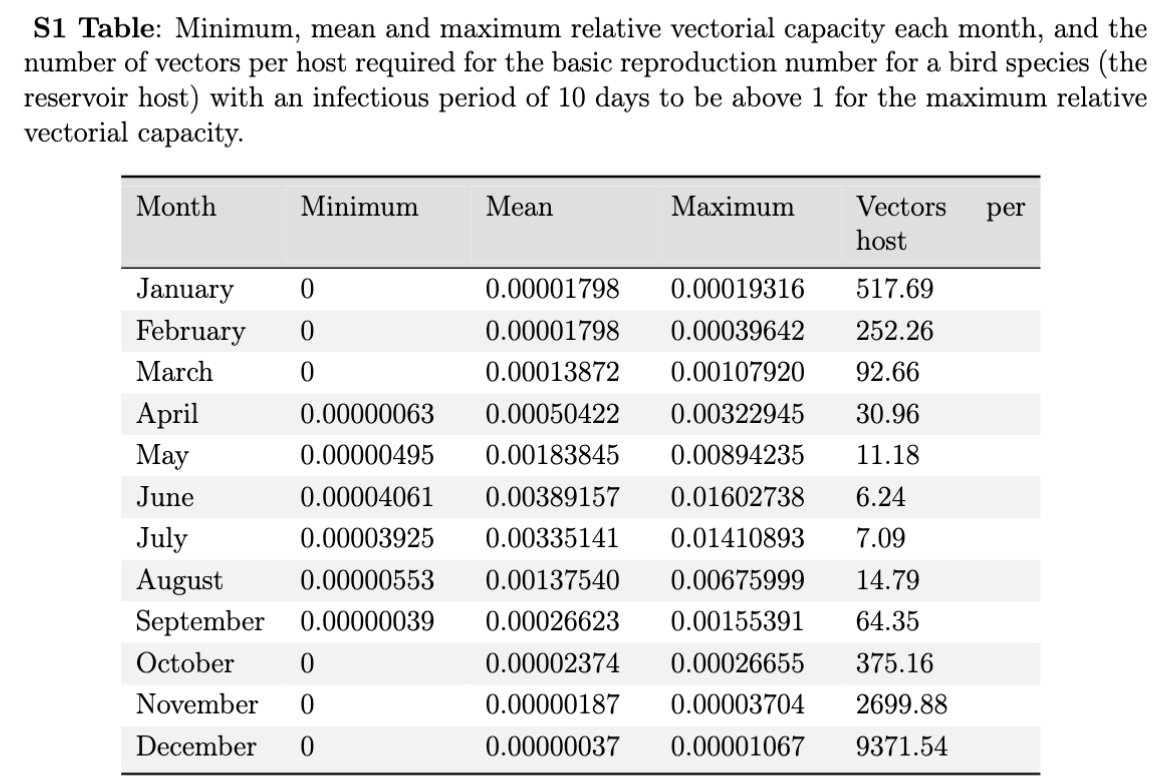

Supplement: S1 Table — (TIFF) [file pcbi.1013359.s002.tiff]
