# Supplementary material for: Quantifying vector diversion effects in zoonotic systems: A modelling framework for arbovirus transmission between reservoir and dead-end hosts
Source: PLoS Comput Biol. 2025 Dec 18;21(12):e1013359. doi: 10.1371/journal.pcbi.1013359 (PMC12795452; doi:10.1371/journal.pcbi.1013359)
Supplement: Attachment — Submitted filename: Response.docx [file pcbi.1013359.s003.docx]

We would like to thank the reviewers for their time reviewing our manuscript. We have responded to the comments below.

Reviewer #1: Article Review – Quantifying vector diversion effects in zoonotic systems: A modelling framework for arbovirus transmission between reservoir and dead-end hosts (Fairbanks, Baylis, Daly, and Tildesley)

Abstract
•Great! Clear, concise and well-written.

Author summary
•Nice. Clear explanation of problem and solution you have found.

Introduction
•Definition of vectorial capacity and relation to basic reproductive number could be made more clear. Can you provide a simple example, equation, or illustration? Or perhaps start from your explanation of the basic reproductive number (the last sentence of the second paragraph), as this is a familiar concept that can be broken down into the parts you define.
Schematic provided and description changed to start from R0. First paragraph now reads:

For vector-borne diseases, the basic reproductive number (the expected number of cases to arise from a single infectious case in a susceptible population) can be expressed as the product of the vectorial capacity (a measure of the potential of a vector population to transmit a pathogen) and duration of the host's infectious period []. Many mathematical models for predicting the risk of disease transmission consider the effects of climate and vector control on the number of vectors [] as a proxy for vectorial capacity. However, vectorial capacity is defined as the total number of potentially infectious bites that would arise from all vectors biting a single infectious host on a single day []. Therefore, vectorial capacity should not only consider the number of vectors, but also their ability to transmit a pathogen, and is therefore the product of both the number of vectors and the relative vector capacity (rVC), defined as the total number of potentially infectious bites that would arise from a single vector biting a single host on a single day (Figure 1).

•For consistency, it looks like you’re missing a line skip between the first and second paragraph.
Now only one paragraph
•At the start of paragraph three, please clarify that you are referring to the one-host limitation from you previous model, and that addressing this limitation is a motivation for your current study.
Added: However, the model only considers how the vectors interact with one type of susceptible host. In this study, we expand this model to address this limitation and consider how the vectors interact with multiple susceptible host species, and how a change in behaviour related to one may influence transmission dynamics to others.
Methods
Model development
•Please find a consistent way to distinguish the two models throughout. For example, “baseline” vs. “extended”, or “single-host” vs. “between-host”/“multiple host”, or something along those lines.
Changed first paragraph of methods to:
The model for simulating the dynamics in a single host species is described in full detail in…
and results to:
Our study extends a single-host mathematical model of the rVC that integrates climatic factors and vector control interventions, introducing interactions between vectors and multiple types of host. This multiple-host model enables…
•It might be helpful to provide the rVC equation in paragraph one.
We appreciate both reviewers' suggestions regarding methodological transparency. To address these concerns while maintaining accessibility for a broad readership, we have added a schematic diagram (Figure 2) that illustrates how the relative vectorial capacity is calculated, showing the key probability components and how composite parameters are derived from underlying scalar and climate-dependent parameters. We believe this visual representation provides the methodological detail requested by Reviewer 2 while offering the clear explanation suggested by Reviewer 1, without overwhelming the main text with extensive mathematical notation. The complete mathematical formulation remains available in the supplementary materials for readers seeking full technical details.

Figure 2: Schematic representation of relative vectorial capacity (rVC) calculation showing the probability of transmission from a reservoir host (h_i) through a vector to another host (h_j). The top panel shows the probability of host to vector transmission on day t, and the bottom panel shows the probability of vector to host transmission on all future days \tau > t. The rVC is the product (×) of these probabilities. Boxes show how composite parameters (solid lines) are calculated from scalar parameters (dashed lines) and temperature-dependent parameters (purple dashed lines), accounting for vector control interventions across multiple host types.
Model application
•Nice, I appreciated your explanation of why a gamma distribution was chosen.
•Please be consistent with use of commas, if you write 26,000, then 2000 should be 2,000.
Added comma
•Great, this section explains the study and assumptions very well.

Results
•Well presented, Figure 2 is especially nice.
•I suggest using a different line style or color for “Disarming” in Figure 4, it’s very difficult to distinguish from “Postprandial killing.” Also, consider adding “due to repellant” to the x-axis label.
Changed.

Discussion
•Include a citation for the “urban heat island effect” in paragraph three.
Citation [44] Thompson, Russell D., and Allen Howard Perry, eds. Applied climatology: principles and practice. Psychology Press, 1997. added
•I might suggest combining paragraphs two and three, there is a lot of information here about temperature that could be condensed. It detracts slightly from your main point and star contributions described in the last three paragraphs.
Done. Now one paragraph:
The optimal temperature for WNV transmission was estimated to be 24.5 C, consistent with previous estimates [refs], representing the thermal optimum where rVC peaks due accelerated mosquito development, shortened gonotrophic cycle, and reduced EIP while maintaining sufficient vector longevity. This optimal temperature falls within the summer temperature range of many temperate regions, including the UK, explaining observed seasonal patterns and suggesting that early warning systems and control strategies would benefit from targeted implementation during the high-transmission windows. Our model predicts higher rVC in urban areas, particularly London, the urban heat island effect creates temperatures 1—3 C higher than rural environments [refs], potentially extending the transmission season in metropolitan areas. However, disease transmission risk depends on both rVC and vector abundance, which is influenced by factors beyond temperature including rainfall, habitat availability and urban landscape heterogeneity [ref],meaning high rVC does not necessarily translate directly to high transmission risk.
•Missing citation in paragraph four: [?]
Fixed

Supplementary Information
•Missing reference after Equation (2)
Fixed

* Be sure to make your code available on GitHub
Will go on <https://github.com/emmafairbanks/EntoModels> with the single-host model. Zenodo will be used to create a unique DOI.

Reviewer #2: The article is well written and logically structured. The analysis appears to represent a novel extension to existing methods. I would like to see several issues addressed before the paper is further considered for publication.

Major concerns:

• Lack of uncertainty analysis and reporting. All key results are reported as point estimates without any consideration of potential uncertainty.
We thank the reviewer for raising this important point about uncertainty. Our objective is to develop a flexible modelling framework and identify general principles that apply across settings, rather than to estimate parameters for a specific location. This study employs a mechanistic model designed to explore system dynamics and develop theoretical insights, rather than fit empirical data with associated statistical uncertainty. Given this framework-oriented nature, we address uncertainty through comprehensive sensitivity analysis rather than traditional statistical confidence intervals, which we believe is the most appropriate approach for this type of work.

**How we address uncertainty in our analysis:**

**Parameter sensitivity across realistic ranges (Figure 5):** Rather than reporting single point estimates, we explicitly vary two key parameters—reduction in biting rate (0-100%) and population coverage (0-100%)—across their full realistic ranges. We additionally present results for three different host selection scenarios (20%, 50%, and 100% of blood meals from reservoir hosts), demonstrating how our conclusions hold across diverse demographic settings. This heatmap approach provides a comprehensive view of how our results vary across parameter space.

**Temperature variability (Figure 3):** We present relative vectorial capacity across a wide temperature range rather than at a single temperature, explicitly showing how transmission potential varies with this critical environmental driver.

**Spatial and temporal heterogeneity (Figure 4):** Our UK-scale simulation incorporates real-world climatic variation across both space (different geographic regions) and time (monthly patterns across 2019-2023), demonstrating how results vary under realistic environmental conditions.

• Insufficient methodological detail is provided in the main text. I suggest content is shifted from the supp material to the main text.
Addressed in highlighted reviewer 1 comment

• Limitations of the analysis are not described in the discussion section and very little reference is made to other similar work.
We respectfully disagree with this assessment and believe the manuscript does address both limitations and contextualisation with existing literature.

Regarding similar work and contextualisation:

Our introduction reviews related work [1-15] to motivate our novel multi-host approach, highlighting that while many models consider vector abundance, they do not consider vectorial capacity or interactions between host types. Throughout the manuscript we reference:

- Previous temperature-dependent transmission models [21, 42] showing our optimal temperature (24.5°C) is consistent with prior estimates
- Our own previous single-host framework [18] that this study extends
- Host selection and blood-feeding pattern research [35, 36, 46, 47]
- Zoonotic disease dynamics [48, 49]

Regarding limitations:

The discussion section currently includes several limitations:

1. "While our model focuses primarily on rVC, it is important to acknowledge that actual disease transmission risk is the product of both rVC and the number of vectors per host abundance. Mosquito population dynamics are influenced by numerous factors beyond temperature, including rainfall patterns, habitat availability and urban landscape heterogeneity [45], meaning high rVC does not necessarily translate directly to high transmission risk."
2. "Host selection of vectors depends on host preference and host availability [18], therefore is likely to vary across settings... Such heterogeneity in host selection across different landscapes significantly impacts WNV transmission potential."
3. "The modes of action of vector control tools change according to the resistance level of vectors and insecticide dose [39, 40]. Insecticide resistance has been reported in Cx. pipiens in Europe [53] and globally [54]."
4. "This modelling framework can be used as an initial indicator of whether these tools are suitable for use on dead-end hosts... However, this may vary across different settings and vector populations [41]."
5. We have additionally added: While our model accounts for heterogeneous vector preferences between host types through the blood index, it assumes spatial homogeneity within each host type, with all individuals of a given species equally likely to be bitten. Spatial clustering of hosts or individual variation in attractiveness could further modulate transmission patterns.

Please provide line numbers on future submissions to assist with the review process.

Other comments:

2.1 Model development

Insufficient methodological details have been provided on this section. At minimum, I suggest that the authors shift the model equations from the supp material into the main text.
Addressed in highlighted reviewer 1 comment.

2.2. Model application

“vector life-span p day” should be “per day”?
Added

“there we set ph->v = 1”. Define this parameter.
Repeat from supplementary so removed.

“…parameterised using Bayesian methods and data from 2145 mosquitoes”. Why state the methods for this study and not the others?
We thank the reviewer for this comment. We mentioned the Bayesian methods for the Vollans et al. [28] study because this parameter estimate comes from a single empirical study, whereas the other parameters (gonotrophic cycle completion rate and vector lifespan) were parameterized by Shocket et al. [21] through systematic review and meta-analysis of multiple studies (four studies for gonotrophic cycle completion [22-25] and three studies for vector lifespan [24, 26, 27]). We felt it was appropriate to highlight the methodological approach for the single-study parameter to provide transparency about how this key parameter was derived, particularly given the large sample size (2145 mosquitoes) and robust statistical framework employed.

“This parameterisation includes the probability of transmission from host to vector and therefore we set ρh→v = 1 for reservoir hosts.” I don’t follow this logic. Why is the probability equal to 1?
Repeat from supplementary so removed. Makes more sense when here.

“Vollans et al. [28] found that the gamma and Weibull distributions performed similarly”. Just confirming that you mean these distributions were equally good fits for the EIP?
Vollans et al. [28] compared multiple distribution models for fitting the extrinsic incubation period (EIP) data and found that the gamma and Weibull distributions had similar Leave-One-Out Information Criterion (LOOIC) values, indicating they were equally good fits to the data.
Changed to: Vollans et al. [28] found that the gamma and Weibull distributions were comparable in their ability to fit the EIP data.

“We calculate the rVC for temperatures between 10 and 32 degrees”. What temperature value? Daily minimum? Daily maximum? Daily mean? Monthly mean?
Here, the temperature is a temperature used to calculate model parameters. This is used to generate a figure to show how the predicted rVCs for each species vary with the temperature input. If using real world data, the user could chose whether to use daily or monthly temperatures, or minimums, maximum or and average.

2.2.2

This is the first mention of the model being applied in the UK. Why is the model applied in the UK? No local cases of WNV have ever been reported in the UK (although it has been found in mosquitoes). I suggest that you add this context to the introduction to motivate your analysis. What are the possible reservoir host species in this setting?
This is stated at the end of the paragraph
“The UK represents an ideal case study for demonstrating applicability of the model, as it encompasses considerable climatic variation across a relatively compact geographical area, allowing for examination of diverse transmission dynamics.”

What do you mean by “simulate” in this section?
To simulate a model means to use a model to predict the behaviour of a real-world system by running computational calculations through the model's equations or rules.

3 Results

3.1 Model development – consider deleting this section. It should be covered in the methods.
The modelling framework itself it a result from this work. It will have any applications and itself is designed to be a useful tool.

3.2 Figure 1 – the short dash and long dash lines are very similar. Please use different indicators.
Changed.

3.4 “In our scenario, where the model is parameterised to describe WNV transmission by Cx. pipiens, we see an increase in vectorial capacity of up to 23.4%.” Given what assumptions about the intervention? These must also be stated.
Changed to:
In our scenario, where the model is parameterised to describe WNV transmission by Cx. pipiens, when dead-end hosts are protected with a purely repellent tool (no killing or disarming effects) at 100% coverage and 100% reduction in biting rate, vectorial capacity among reservoir hosts increases by up to 23.4%.

Figure 3 caption. “When a tool is used by…” Should this be when a tool is “applied to”?
Changed

“Percentage of blood meals taken from reservoir host” – this not described in the Figure 3 caption – please add.
Added: Columns show three scenarios representing the percentage of blood meals taken from reservoir hosts (birds): 20\%, 50\%, and 100\%.

Figure 4: Again, short and long dash lines are too similar. Please change.
Changed.

4. Discussion

“Independently” – what do you mean by this?
Removed

“in line with previous estimates”. Please elaborate here. What did these studies find? What was their approach? How it is similar/different from your approach?
In line with reviewer 1 comments this section has been significantly reduced. The appropriate studies are cited, such that authors wanting to know more about other methods can find the relevant information.

“Many health agencies suggest using repellent to reduce the risk of vector-borne disease [48–50]]. This work shows that for zoonotic diseases this may not always be the case.” The use of “this” in sentence two is confusing. Consider “our study” and re-state the finding that you refer to.
Done.
Also, I would be very careful here.
This paragraph continues:
More investigation is needed into the effects of increasing vectorial capacity on disease transmission. … This modelling framework can be used as an initial indicator of whether these tools are suitable for use on dead-end hosts, and suggest target product profiles of additional tools for use.
While you show that rVC can increase when repellent is used by human dead-end hosts, the individuals using repellent are still protected from disease exposure.
Also worth considering the important limitations of your work here. You have only considered two hosts and the effect of repellent use in one of those hosts. What about the impact of other wildlife dead-hosts? Bites could be diverted to those hosts instead of the reservoir hosts (i.e. zooprophylaxis). Please discuss.
This is already allowed for in the model by adjusting by blood indices.

“This modelling framework is flexible for application to other vectors and pathogens.” Can you say a little more about how your model framework would be applied to the two example systems listed?
Added: Parameterising the model for these pathogens could lead to further exploration of these dynamics.

“This modelling framework can be used to assess whether these tools are suitable
for use on dead-end hosts, and suggest target product profiles of additional tools for use.” I don’t follow this point, can you say more here?
This is illustrated through application in section 3.5 of the results.

Supp methods

?? – some citations are missing
Fixed as per reviewer 1 comment.

Beta is not described.
Added: The composite parameter $\beta$ represents the adjusted rate of host-encountering events, accounting for baseline host selection and the effects of vector control tools across all host types.

Equation 3 and Table S2. How are day s and day t different? I got a little confused here.
Day t is a specific time point within the modelling framework - the day we are calculating the rVC for. This is always fixed. We use s in the formula on days which are not specifically t or tau.

“…considered the climatic dependence of the rates of gonotrophic cycle completion, vector mortality and pathogen extrinsic incubation period (EIP) completion”. You also consider these dependencies in your work, please described them. Or at least point to section 1.2.1?
Addressed in next comment.

What “temp” was used for parameterisation in equations 11, 12, and 13?
Added “where temp is the temperature”
